# Multiple Pregnancy after Gonadotropin-Intrauterine Insemination: An Unavoidable Event?

**DOI:** 10.5402/2011/465483

**Published:** 2011-12-29

**Authors:** Shirley A. Fong, Vidya Palta, Cheongeun Oh, Michael M. Cho, Jacquelyn S. Loughlin, Peter G. McGovern

**Affiliations:** ^1^Department of Obstetrics, Gynecology, and Women's Health, New Jersey Medical School, 185 South Orange Avenue, MSB E506, Newark, NJ 07103, USA; ^2^Division of Biostatistics, New York University School of Medicine, 650 First Avenue Room 556/558, New York, NY 10016, USA

## Abstract

*Objective*. Determine which factors predict multiple pregnancy in gonadotropin-intrauterine insemination cycles so that cancellation criteria might be developed. *Study Design*. Retrospective chart review of all patients undergoing gonadotropin-intrauterine insemination over a continuous 36 month period. *Results*. No factors examined were able to predict the occurrence of multiple pregnancy. *Conclusion*. Multiple pregnancy is an unavoidable complication of gonadotropin-intrauterine insemination treatment.

## 1. Introduction

Artificial insemination is an effective treatment for subfertility, originally described in 1957 by Mastroianni [1]. Inseminations are commonly accompanied by ovulation stimulation, which increase the pregnancy but markedly elevate the risk of multiple birth. In a multicenter randomized trial, Guzick and colleagues [2] reported pregnancy rates per insemination cycle of 2% with intracervical insemination (ICI), 5% with intrauterine insemination (IUI), 4% with gonadotropin-ICI, and 9% with gonadotropin-IUI. Thus, even gonadotropin with ICI was less efficacious than IUI alone.

In 2004 [3], the Centers for Disease Control reported that the twin birth rate was 3% at 32.3 per 1000 total live births, a high record. This is a 71% increase since 1980 when the rate was 18.9 per 1000 total live births. In a study of higher-order multiples in 1997 [4], it was found that 43% were from assisted reproductive technology, 38% were from ovulation inducing drugs, and 20% were spontaneous. Concerns about obstetrical and neonatal risks associated with multiple pregnancy suggest the need to develop better predictors of multiple gestation.

Improvements in IVF have led to increasing pregnancy rates at the same time that the number of embryos transferred has declined. The American Society for Reproductive Medicine has issued practice guidelines [5] on the number of embryos suggested for transfer in IVF. Together, these forces have led to a drastic reduction in high-order multiple births after IVF. In the most recent SART report [6] (2009 National Summary Data, http://www.sart.org/), less than 2% of all IVF births involve 3 or more babies. The same practices cannot be used to limit multiple birth after gonadotropin-IUI treatments [7]. Despite its greater associated risks, gonadotropin-IUI has remained a popular treatment option since it is more affordable than IVF for many couples. 

Physicians have long sought ways to predict pregnancy and multiple gestations during gonadotropin-IUI. Obvious factors are number of follicles and peak estradiol. Peak estradiol should be a true reflection of the entire developing follicular cohort, both large- and intermediate-sized follicles. Some authors [8, 9] have found a correlation between peak estradiol and the number of follicles while others have not [11]. Adding to the confusion are significant differences between studies in terms of which follicle sizes they have considered “large” and whether they have used mean or largest diameter. In addition, there are variations in follicle size measurements both within and between observers, along with variations in image quality and resolution between ultrasound machines.

Using our gonadotropin-IUI database, we sought to determine which patient-specific and cycle-specific factors were predictive of high-order (three or more sacs) multiple and any (two or more sacs) multiple pregnancy.

## 2. Materials and Methods

IRB approved retrospective chart review of all gonadotropin-IUI cycles at the New Jersey Medical School Center for Reproductive Medicine from January 1, 1998, through December 31, 2000. 861 gonadotropin-IUI cycles were performed on 428 patients. No cycles were excluded from analysis. Our hypothesis was that number of mature follicles and/or peak estradiol level would be most predictive of multiple pregnancy after gonadotropin-IUI therapy.

A baseline transvaginal ultrasound was performed on cycle days 1, 2, or 3 to exclude residual cysts prior to initiating treatment. Ovulation induction using gonadotropins began on day 3 of a spontaneous menstrual cycle or a progestin-induced withdrawal bleed. Starting dose of gonadotropins was determined by the physician. During this period, both recombinant and urinary gonadotropins were used depending on physician preference and product availability. Transvaginal ultrasound was performed and estradiol level drawn after three to five nights of medication. Ultrasounds and estradiol levels were repeated as clinically indicated. Medications were adjusted as needed. Follicle size was determined by transvaginal ultrasound taking two measurements perpendicular to each other and recording the average in millimeters. When one or more follicles reached a mean diameter of ≥16 mm, hCG 5,000 IU was given intramuscularly. Single IUI was performed 38 hours after hCG. hCG 2,500 IU was administered intramuscularly four days after the IUI for luteal support. No supplemental progesterone was given. Serum *β*-hCG was drawn 2 weeks after insemination.

Main outcome measure was the occurrence of multiple pregnancy (≥3 versus ≥2 versus one gestational sac) in resulting clinical pregnancies. Patients were classified according to their principle infertility diagnosis: male factor, tubal factor, endometriosis, ovulatory dysfunction, uterine factor, or unexplained infertility. Ovulatory dysfunction was further broken down into oligoovulation or anovulation due to hypogonadotropic hypogonadism (WHO I), polycystic ovary syndrome (WHO II), or diminished ovarian reserve (WHO III). Male partners generally had at least 3 million motile sperm in their processed specimens. Tubal patency was confirmed by laparoscopy or hysterosalpingogram. At least one documented open fallopian tube was necessary. Endometriosis was diagnosed at laparoscopy and classified according to the American Society of Reproductive Medicine (1996) classification system. Assessment of ovulation was based on history and midluteal serum progesterone. A midluteal serum progesterone >5 ng/mL was considered ovulatory. Patients with uterine factor had a history of a uterine defect which was repaired by either operative hysteroscopy or open laparotomy prior to undergoing treatment. Unexplained infertility was diagnosed when there was no abnormality found in the infertility evaluation of both partners.

Patient-specific and cycle-specific parameters were examined. Patient-specific parameters included were female age, duration of infertility, infertility diagnosis, highest basal FSH level, previous use of clomiphene citrate, gonadotropins or IVF, parity, ovulatory status, lowest Kruger's morphology, and use of husband versus donor sperm. Cycle-specific parameters included were stimulation protocol, type of gonadotropin used (recombinant, urinary, or combination), days of gonadotropin stimulation, total ampoules of gonadotropin used, maximum daily dose of gonadotropins, number of mature follicles, TNMC inseminated, maximum endometrial thickness, and peak estradiol level.

Multiple logistic regression was used to evaluate predictors of any multiple clinical pregnancy (2 or more gestational sacs) or multiple high-order clinical pregnancy (3 or more sacs). Sigma Stat (Version 2.0, 1997, SPSS Inc., Chicago, IL, USA) was used for calculations. *P* value <0.05 was considered statistically significant.

## 3. Results

428 women aged 18–46 underwent 861 gonadotropin-IUI cycles, resulting in 95 live births (11.0% per cycle). The clinical pregnancy rate was 13.8% per cycle (119/861). Patient- and cycle-specific parameters associated with the occurrence of live birth pregnancy were examined (Table 1). We found that younger age, fewer years of infertility and higher peak estradiol levels all predicted success, but that no other factors were significantly different between the two groups. Of the 95 pregnancies which ultimately resulted in a livebirth (Table 2), at the end of the first trimester 4 were quadruplets (4.2%), 7 were triplets (7.4%), 17 were twins (17.9%), and 67 were singleton (70.5%). Of the high-order multiple pregnancies (*n* = 11 or 11.6%), 8 underwent elective fetal reduction (7 to twins and 1 to a singleton), and all delivered viable children. Three attempted to carry: two as triplets (both women delivered viable triplets, but one child died within 24 hours) and one as quadruplets (delivered 4 living children at 22 weeks but none survived). Thus, in our small series, neonatal outcomes in those women who did not undergo elective fetal reduction were not good.

All cycle and patient parameters in our database were investigated as predictors of multiple clinical pregnancy by looking at cycles which resulted in 1 gestational sac versus those which resulted in ≥2 or ≥3 gestational sacs. None of the patient- or cycle-specific parameters examined (including age and number of mature follicles) were significant predictors of the occurrence of any (≥2) or high-order (≥3) multiple pregnancy (*P* > 0.05).

For illustrative purposes, we present our data for some factors which seemed likely (based upon previous studies) to be useful predictors of multiple pregnancy. We hypothesized that perhaps we might find a useful cutoff for cycle cancellation to limit the occurrence of high-order multiple pregnancy (3 or more sacs). It has been suggested that one may cancel or convert to IVF those gonadotropin-IUI cycles which seem to be at elevated risk and therefore prevent or at least limit the occurrence of high-order multiple pregnancy.  Table 3 demonstrates the relationship between several factors which have [10] been proposed to limit the occurrence of high-order multiple pregnancy and the development of high-order clinical pregnancy (≥3 sacs) in our series. [Since single sac clinical pregnancies are more likely than multiple sac gestations to undergo spontaneous miscarriage, the high-order multiple pregnancy rate per clinical pregnancy (9.2%) is lower (11/119 clinical pregnancies contained 3 or more sacs) as compared to the high-order multiple pregnancy rate (11.5%) expressed as a percentage of ongoing or live birth pregnancies (11/95 ongoing pregnancies had 3 or more sacs).

We can evaluate the utility of some of these criteria for cycle cancellation in our couples. For example, in our series, 102/861 or 11.8% of cycles developed ≥5 mature follicles. Theoretically, if all of these cycles had all been cancelled or converted into IVF due to excessive ovarian response, 8 clinical pregnancies would have been prevented; 3 of which included ≥3 sacs. The remaining 759 treatment cycles in which ≤4 mature developed would have produced 111 clinical pregnancies, of which 8 (7.2%) would have included ≥3 sacs. Cancellation of those 102 cycles (nearly 12% of cycles) would have only slightly lowered (9.2 to 7.2%) the clinical high-order multiple pregnancy rate, which would remain at 7.2%. Unfortunately, this risk of high-order multiple pregnancy remains unacceptably high.

Instead of follicle number, we can try using peak estradiol level In our series, 133/861 or 15.4% of cycles developed a peak estradiol level ≥1200 pg/mL. Theoretically, if all of these cycles had all been cancelled or converted into IVF due to excessive ovarian response, 25 clinical pregnancies would have been prevented: 4 of which included ≥3 sacs. The remaining 728 treatment cycles in which the peak estradiol level was below 1200 pg/mL would have resulted in 94 clinical pregnancies, of which 7 (7.4%) would still have included ≥3 sacs. Unfortunately, this risk of high-order multiple pregnancy remains unacceptably high. Review of Table 3 clearly demonstrates that no reasonable criteria can be found which will reduce the occurrence of high-order multiple pregnancy to acceptable levels and that these extremely complicated pregnancies remain an unavoidable side effect of gonadotropin-IUI treatment.

## 4. Discussion

Our clinical pregnancy (13.8%) and live birth (11.0%) rates are consistent with published data from other trials [2, 12–15]. Although many trials have looked at clinical pregnancy rates after gonadotropin-IUI treatments, few studies have reported live birth rates—even though live birth is the primary outcome of interest and therefore the most relevant information for patient counseling.

The risk of high-order multiple pregnancy in our series (11/95 or 11.6% of ongoing pregnancies were triplets or quadruplets) was notable. Previous investigators [2, 7] document similar risks, ranging from 7 to 10%. The dramatic increase in obstetrical and neonatal risks associates with the presence of high-order multiple pregnancies is well known and necessitates careful patient counseling before embarking upon gonadotropin-IUI therapy.

We found that the occurrence of multiple pregnancy could not be predicted by any of the factors examined. In our series, the number of follicles measured was not helpful in predicting multiple pregnancy. Several previous investigators have found that the number of follicles predicted multiple pregnancy, although the critical size for the follicles in these studies varied from 12 to 15 mm. [6, 7, 17]. Dickey et al. [7] reported on over 3600 IUI cycles and noted a rate of 3 or more gestational sacs of 7.2%. They noted that different factors were critical in predicting the occurrence of high-order multiple pregnancy at different ages: under age 35, the number of follicles ≥15 mm was the best predictor of high-order multiple pregnancy (and serum estradiol was not useful), while in women aged 35–42, serum estradiol level over 1,000 pg/mL was the most useful predictor (and number of follicles was not helpful). Our results confirm and extend those of Gleicher et al. [16], who noted that peak serum estradiol level and/or number of preovulatory follicles were not useful in predicting/preventing high-order multiple pregnancy after gonadotropin-IUI. They suggested that there is “… an unacceptably high incidence of high-order multiple pregnancies after the induction of ovulation with gonadotropins.” And also that “… better criteria cannot easily be developed without negatively affecting overall pregnancy rates.” Unlike these authors, our study looked at many other parameters beside estradiol and follicle number, yet we were still unable to determine safe settings for cycle cancellation which would prevent an unacceptable level of high-order multiple pregnancies after this treatment.

Dickey published an updated analysis in 2009 [10], including references from 130 articles, and developed several conclusions. Risk factors for high-order multiple pregnancy included ≥7 preovulatory follicles (≥10–12 mm), E2 > 1,000 pg/mL, early cycles of treatment, age < 32, low BMI, and use of donor sperm. Unfortunately, cancellation of any treatment cycle in which any one of these many criteria occur would lead to very few completed cycles and would be counterproductive to therapeutic relationship. The author did not provide specifics as to what combinations of these risk factors could be used to establish reasonable cancellation criteria. Based upon our analysis, we could determine no clear criteria which may be used to avoid high-order multiple pregnancy which will not lead to the cancellation of an unacceptably high % of treatment cycles.

Dickey [10] also claimed that age ≥ 38 was a low-risk factor. Since our series included only 3 women ≥ 35 years with ≥ 3 sacs, separate statistical analyses by female age were not possible. But along with a lower rate of multiple pregnancy, there was a much lower efficacy in terms of pregnancy success. It can be seen that high-order multiple pregnancy rates declined along with overall clinical pregnancy rates: ≤34 years 18.6% clinical pregnancies per IUI cycle and 12.7% ≥ 3 sacs, at 35–37 years 13.7% clinical pregnancies per IUI cycle and 6.0% ≥ 3 sacs, and at ≥38 years 8.1% clinical pregnancies per IUI cycle and 4.3% ≥ 3 sacs. Even in the older age groups, the rate of high-order clinical pregnancies remains notable at 4.3%. Thus even in the “older” group with a reduced chance of pregnancy success, the rate of high-order multiple pregnancy remained more than double the IVF triplet rate.

## 5. Conclusion

Gonadotropin-IUI carries a significant risk of high-order multiple birth (11.6%) among resulting viable pregnancies. Cancellation of cycles in which elevated estradiol levels (>1200 pg/mL) or an excessive number of follicles (≥5) develop would not reduce this risk to acceptable levels.

## Figures and Tables

**Table 1:** Patient- and cycle-specific parameters in live birth and no live birth groups, mean (95% CI).

	Live birth	No live birth	*P*
Female age (yrs)	33.2 (32.4–34.1)	35.7 (35.3–36.0)	<0.001
Years of infertility	2.3 (1.9–2.8)	3.3 (3.0–3.6)	0.038
Basal FSH (IU/L)	8.3 (7.4–9.2)	8.4 (8.1–8.7)	NS
Kruger (%)	11.0 (9.7–12.3)	10.7 (10.4–11.1)	NS
Days of Gn.	9.4 (8.9–10.0)	9.1 (8.9–9.3)	NS
Total Gn. (amp)	26.6 (23.4–29.7)	29.1 (27.9–30.3)	NS
Max daily Gn. (amp)	2.9 (2.6–3.2)	3.5 (3.2–3.8)	NS
No. of follicles	2.4 (2.2–2.7)	2.6 (2.5–2.7)	NS
TNMC (×10^6^)	69.5 (54.9–84.1)	78.7 (72.2–85.2)	NS
Max. EM thickness (mm)	10.7 (10.2–11.3)	10.1 (9.9–10.2)	NS
Peak E2 (pg/mL)	835.0 (737.0–933.0)	687.1 (654.0–720.1)	0.005

**Table 2:** Obstetrical outcome in live birth pregnancies by plurality.

Plurality	*n*	Obstetrical management	Birth outcomes
Quads	4	1 carried 3 elective fetal reduction to twins	All lost at 20 wks all live births
Triplets	7	2 carried 5 elective fetal reduction (4 to twins, 1 to singleton)	1 delivered viable triplets, 1 delivered triplets, 2 survived 4 delivered twins, 1 delivered singleton
Twins	17	17 carried	15 delivered viable twins 2 spontaneously reduced to singleton
Singleton	67	All carried	All singleton

**Table 3:** Cancellation criteria to prevent high-order multiple pregnancy (≥3 sacs) during gonadotropin-IUI cycles.

	Clinical pregnancy *n* = 119	Cycles *n* = 861	≥2 sacs *n* = 30	≥2 sacs per clinical pregnancy (%)	≥3 sacs *n* = 11	≥3 sacs per clinical pregnancy (%)
No. of mature follicles						
1-2	68	490	15	15/68 (22.0%)	4	4/68 (5.8%)
3	23	173	3	3/23 (13.0%)	2	2/23 (8.6%)
4	20	96	6	6/20 (30.0%)	2	2/20 (10.0%)
≥5	8	102	6	6/8 (75.0%)	3	3/8 (37.5%)

Peak E2 (pg/mL)						
≤800	62	558	11	11/62 (17.7%)	4	4/62 (6.4%)
801–1000	15	101	5	5/15 (33.3%)	2	2/15 (13.3%)
1001–1200	17	69	4	4/17 (23.5%)	1	1/17 (5.8%)
1201–1500	13	75	3	3/13 (23.0%)	3	3/13 (23.0%)
≥1500	12	58	7	7/12 (58.3%)	1	1/12 (8.3%)

Age						
≤34	63	338	18	18/63 (28.6%)	8	8/63 (12.7%)
35–37	33	240	8	8/33 (24.2%)	2	2/33 (6.0%)
38–40	16	156	3	3/16 (18.7%)	0	0/20 (0%)
41-42	4	78	1	1/4 (25.0%)	1	1/4 (25.0%)
≥43	3	49	0	0/3 (0%)	0	0/3 (0%)

Max. daily gonadotropin dose						
≥5	18	194	2	2/18 (11.1%)	0	0/18 (0%)
4	17	129	3	3/17 (17.6%)	2	2/17 (11.7%)
3	32	186	7	7/32 (21.8%)	4	4/32 (12.5%
≤2.5	52	352	18	18/52 (34.6%)	5	5/52 (9.6%)
